# Locally Delivered Antibiotics in Fracture-Related Infection

**DOI:** 10.7759/cureus.73210

**Published:** 2024-11-07

**Authors:** Henry Mills, Liam Donnelly, Simon Platt

**Affiliations:** 1 Orthopaedics, Gold Coast University Hospital, Gold Coast, AUS; 2 Trauma and Orthopaedics, London North West University Healthcare National Health Service Trust, London, GBR

**Keywords:** carriers, ceramics, fracture-related infection, hydrogels, local antibiotics, orthopaedic trauma, polymethyl methacrylate (pmma), vancomycin powder

## Abstract

The prevention and treatment of fracture-related infections (FRIs) pose significant challenges in orthopaedic trauma care, with current practices predominantly relying on systemic antibiotic administration. However, locally delivered antibiotics achieve substantially higher tissue concentrations and minimise systemic side effects. Whilst extensively researched in periprosthetic joint infections (PJIs), the use of local delivery methods is increasingly prevalent in FRI prevention and treatment. Various local delivery methods such as powders, aqueous injections and carriers such as cement, bone graft, bioceramics, polymers and hydrogels have been explored. Biodegradable antibiotic carriers offer a promising alternative to non-absorbable carriers (i.e., cement), which necessitate surgical removal. There is good evidence for the use of local antibiotics in preventing and treating FRI, particularly in high-risk fractures or in treating more severe, resistant infections. Despite theoretical concerns, reports of adverse events in human studies are rare. To enhance our understanding of the safety and efficacy of these methods across various fracture patterns, further prospective randomised controlled trials are warranted. This article describes the current strategies and methodologies for FRI prevention and treatment and reviews the existing evidence base.

## Introduction and background

Fracture-related infection (FRI) is a serious complication of fracture and represents a significant challenge in trauma surgery. In 2018, a definition was provided by the FRI consensus group, which has since been updated in 2020 [1,2]. It consists of a flow chart of confirmatory and suggestive criteria, which can be identified at medical history and clinical examination and also at surgical exploration. The incidence ranges from 1% in closed fractures to 30% in Gustilo-Anderson (GA) type 3 open tibial fractures [3]. Patient factors, such as immunocompromise, obesity, socioeconomic status, hygiene, vascular disease and smoking status, increase the risk [4]. Patient consequences of FRI include prolonged healing, non-union, ongoing morbidity and mortality. Infection recurrence and amputation represent notable complications, occurring in 9% and 3% of patients, respectively [5]. Patients with successfully treated long bone FRI have significantly poorer physical, mental and overall health scores over four years of follow-up post-final surgery [6]. There is also an increased financial burden on the health service provider. Direct hospital costs from longer hospital stays, readmissions and increased operating time have been recorded at eight times higher than for non-FRI patients [7]. However, this does not account for social care costs, transportation fees and the price of labour.

Despite the cost and morbidity, guidelines for managing FRI are limited. This is partly due to the lack of a consensus definition for FRI until recently, with most published studies using heterogeneous definitions, which preclude comparison [4,5,8,9]. Furthermore, FRI encompasses a vast range of clinical presentations and severity, which complicates management. A new classification system based on fracture healing potential, patient factors and soft tissue impairment was published in 2024 to aid with categorising these cases [10]. The current standard preventative measures for FRI will involve intravenous (IV) antibiotics. Either an extended course for an open fracture or an induction dose intraoperatively for closed fractures. In established FRI, there is a range of evidence [9]. Most regimes will include systemic antibiotics with the goal of either infection eradication or suppression of infection until union. Implant exchange may be advised in cases of later-onset infection. After union, the implant can be removed and is usually followed by six weeks of systemic antibiotics for osteomyelitis treatment [9].

Systemic therapy is rarely successful, particularly in cases of biofilm formation [9]. Locally delivered antibiotics have been integral to managing prosthetic joint infection (PJI) for many years and are now becoming more prevalent in the prevention and treatment of FRI due to the emergence of promising results, albeit with a lack of high-quality evidence [11-13]. This review will discuss the rationale and methods for the use of local antibiotics in fracture surgery, the various delivery systems and their evidence for preventing and treating FRI.

## Review

Rationale

Compared with systemic delivery, locally administered antibiotics will achieve much higher local concentrations without elevating serum levels and, therefore, yield a more favourable side effect profile [14,15]. This concept is amplified in a fracture as the vascular supply to the local tissue is compromised by the anatomical insult [16].

The treatment efficacy of bacterial infection is influenced by the concentration levels of the antibiotic at the site of infection [13]. In cases of planktonic infection, the minimum inhibitory concentration (MIC) needs to be reached. However, the development of a biofilm - a complex of microbial communities encased in the extracellular polymeric matrix - necessitates a higher concentration to successfully eradicate infection; the eradication of biofilm may not be solely dependent on antibiotic concentration alone. The minimum biofilm eradication concentration (MBEC) is many magnitudes higher than the MIC. For example, vancomycin-sensitive *Staphylococcus aureus* may have an MBEC of over 1000 μg/mL compared to its planktonic MIC of 2 μg/mL [17,18]. Unlike local delivery, systemic therapy alone cannot achieve the MBEC [4,9,19]. Potential benefits of higher local concentration include lower infection rates, higher rates of union and infection eradication in established FRI, and scope for treating resistant and biofilm infections [4,20].

Other benefits include dead space management, structural support and improved antibiotic compliance and stewardship [3,21,22]. Dead spaces, often encountered after a thorough debridement, provide the ideal environment for bacterial growth. Local antibiotic carriers can be shaped to occupy these high-risk zones [21]. Local delivery also evades the issue of noncompliance. Noncompliance is a known issue associated with oral antibiotic use, with fear of adverse effects being reported as the most significant contributing factor [22].

Considerations

Local delivery of antibiotics in high concentration is generally considered safe [20]. However, concerns around toxicity, resistance and systemic side effects have been raised.

Osteocyte toxicity has been observed in in-vitro studies. The concentrations at which osteoblast cell number and osteogenic activity are significantly impacted vary greatly depending on the antibiotic [23-26]. Gentamicin was noted to reduce cell number and osteogenic activity by >25% at <200 μg/mL, whereas a <25% effect was seen at concentrations of >2000 μg/mL of vancomycin [23]. However, animal studies investigating high-dose vancomycin powder, tobramycin powder and gentamicin polymeric coated intramedullary nails in-vivo have shown no insult to bone on microradiographs, histology or biomechanic testing compared with placebo [27-29]. Furthermore, increases in delayed or non-union rates have not been identified in human studies [16,30]. However, these human trials were not designed to assess the cytotoxic effects of antibiotics. Further in-vivo studies are required to determine the relevance of cytotoxicity if union rates are unaffected or improved with local antibiotics. 

The generation of resistant bacteria is also a concern. Vancomycin is not considered a first-line antibiotic in the treatment of the most prevalent species in postoperative infection (*Staphylococcus*), yet it has been suggested for prophylactic use and, therefore, could potentially lead to the formation of new vancomycin-resistant *Staphylococcus aureus* (VRSA) [12,31]. Furthermore, concern exists for sustained subtherapeutic release from carriers. Gentamicin-resistant strains have been cultured from gentamicin-loaded beads from 90% of patients after revision surgery for PJI [32]. However, some authors suggest that more effective prophylactic antibiotic regimens would reduce the current rate of emerging strains in orthopaedics seen with routinely used systemic antibiotics [20]. In theory, lower systemic antibiotic concentrations for shorter durations associated with local antibiotics would be expected to produce fewer resistant bacteria relative to systemic delivery [12].

Finally, some authors have suggested an association between antibiotic cement spacers and acute kidney injury (AKI) in PJI revision surgery [33,34]. However, these operations are complex, and most evidence shows significant associations with independent risk factors for AKI, such as perfusion-related issues, pre-existing kidney disease and comorbidity, which are frequent in this cohort [35,36]. The concern for systemic side effects with vancomycin powder is much lower as serum absorption levels have been recorded as negligible [16,37].

Local antibiotics should, therefore, be approached with caution, especially prophylactic use. An awareness of the potential for adverse events should be considered.

Local antibiotic delivery systems

Local antibiotic delivery systems can be categorised into those without a carrier and those with a carrier, which can be absorbable or non-absorbable. Vancomycin and aminoglycosides are the most commonly used locally delivered antibiotics. They provide a broad spectrum of efficacy, have extremely low rates of anaphylaxis and have good compatibility with carriers [38]. Gentamicin has bactericidal properties in high concentrations, making it effective for resistant bacteria, such as methicillin-resistant *Staphylococcus aureus* (MRSA) and biofilm [39].

Naked antibiotic delivery

Antibiotics without a carrier, also known as ‘naked’ antibiotics, are delivered in either aqueous or powder form. They are cheaper, but the duration of antibiotic release is limited [3,12]. Johnson et al. measured serum and drain output antibiotic concentrations from patients receiving intra-articular vancomycin powder during primary total joint arthroplasty. All serum measurements were subtherapeutic (<15 μg/mL). Drain concentrations, however, peaked three hours post-operation at 922 μg/mL before declining at a half-life of 7.2 hours and were thought to be subtherapeutic by 64 hours. However, the minimum time required for clinical efficacy is unknown [14].

Prevention

The use of naked antibiotics in fracture was first described in 1939 by Jenson et al., who placed sulfanilamide powder into 39 compound wound fractures post-debridement and recorded no primary wound infections [40]. Vancomycin powder and aqueous aminoglycoside injections have since emerged as more frequently used prophylactic local antibiotics in orthopaedic surgery [3,12]. Reduced rates of infection have been reported with vancomycin powder in spinal surgery [41,42]. However, recent meta-analyses of its use in primary arthroplasty surgery have been conflicting [43,44]. Aqueous gentamicin has shown lower bacterial wound counts in animal studies when compared with systemic cefazolin and lower rates of infection in total shoulder replacement surgery [20,45].

In fracture patients, various retrospective studies have reported on the effects of vancomycin powder [46-51]. Qadir et al. recorded zero infections for 35 patients with high-risk calcaneus, pilon or tibial plateau fractures treated with combined systemic antibiotics and powdered vancomycin [48]. This was significantly lower than the 58 infections recorded in the 548 patients treated with systemic therapy alone (p = 0.04). Wang et al. found lower rates in high-risk tibial plateau fractures (p = 0.038). Although these studies matched the groups for important variables such as age, diabetes, tobacco use, polytrauma, fracture classification and post-operative systemic antibiotic duration, they were performed at single centres with vancomycin use being at the discretion of the surgeon and are therefore prone to selection bias [51]. Owen et al. studied the combined use of vancomycin with tobramycin powders in high-energy acetabular and pelvic ring fractures. They reported a statistically significant 90% reduction in infection rates for cases with less than 1 L of blood loss (OR = 0.1 (0.01-0.8)). However, there was heterogeneity between the groups with higher rates of complex acetabular fracture in the control group [47]. Conversely, other retrospective studies have failed to identify any benefit in infection rates [46,49,50]. Vaida et al. reported no significant benefit in open lower limb fractures [50]. Singh et al. found no advantage in staged fixation after high-energy tibial plateau and pilon fractures [49]. However, both of these studies were underpowered, involving 46 and 10 patients in the treatment groups, respectively. A larger study by Cichos et al. found no significant reduction in deep and superficial infection rates when vancomycin or combined vancomycin and tobramycin powder were used in 463 open reduction and internal fixations for acetabular fractures [46]. To the authors’ knowledge, two randomised controlled trials (RCTs) have been conducted to assess the prophylactic use of vancomycin powder in FRI [16,52]. Gandhi et al. conducted an RCT on 88 patients with closed fractures of predominantly Tscherne grade 0. There was a non-significant lower rate of infection at six weeks in patients treated with vancomycin powder combined with systemic antibiotics compared with systemic antibiotics alone (n = 0/44 vs. n = 2/44). However, demonstrating a statistically significant difference in a small cohort of low-risk fractures would be difficult [52]. The most thorough RCT was conducted by O’Toole et al., who included 980 patients with tibial articular fractures at high risk for infection. Vancomycin powder conferred a lower risk of gram-positive deep surgical site infection within 182 days of surgery, with a risk reduction of 3.7% (p = 0.02). There was no significant difference in rates of deep gram-negative infection or superficial infection [16].

Fewer studies exist for local aminoglycoside use. Lawing et al. retrospectively reviewed 168 open fractures receiving local aminoglycoside injection and found significantly lower rates of both superficial (p = 0.015) and deep infections (p = 0.034) than the control group. However, the study contains multiple fracture patterns of both upper and lower extremities, and although the groups were matched statistically, the study was subject to surgeon bias [30].

As discussed, most studies are retrospective and have limitations. The results vary depending on factors such as the severity and pattern of the fracture and study design. The use of prophylactic local antibiotics remains controversial, with benefits most likely to be with high-risk fractures. Further RCTs on individual fracture types would further validate findings.

Treatment

Implant retention is desirable in FRI treatment to facilitate union. Treatment, therefore, aims to control the infection until union is achieved, either by eradication or suppression. In severe cases, biofilm suppression is necessary. Unfortunately, naked local antibiotics have a short therapeutic window, and thus, research into their use in FRI treatment is not well evidenced. To maintain therapeutic levels, frequent injections or dosing via catheters would be required. Such techniques have been described in single-stage revision for PJI with high success rates in small retrospective series of resistant infections [19,53]. In fractures, a continuous local antibiotic perfusion (CLAP) system has been developed in Japan for severe FRI cases [39,54,55]. The concept involves antibiotic administration through intramedullary needles and double-lumen tubes into the soft tissue around the infection site. Leachate is drained through the tubes or a negative pressure wound dressing. Gentamicin was used for its bactericidal properties, which are effective against resistant bacteria, such as MRSA and biofilm, as well as for the ease of serum monitoring. The therapy lasted for an average of nine to 17 days between the studies. Results were promising, with near 100% success rates and a lack of side effects. However, the studies are limited by small numbers and no comparison groups. Furthermore, the technique is not perfect because it requires invasive intramedullary needles and drainage tubes for a number of weeks. Its value may be reserved for more severe or refractory cases.

An animal study by Wei et al. reported infection eradication after a single-stage revision for MRSA PJI with intrawound vancomycin powder and two weeks of systemic vancomycin [56]. It is possible that this application of vancomycin powder could be adapted to FRI treatment when the implant is for exchange.

Carriers

The primary advantages of carriers include prolongation of antimicrobial half-life and antibiotic elution control. Optimal release extends the therapeutic release until completion in order to minimise subtherapeutic dosing. Cement (or polymethylmethacrylate (PMMA)) has been the most frequently used carrier since its conception in the 1970s [12]. However, it is non-absorbable and requires further operations for removal as it could become a nidus for infection. Interest in absorbable carriers is growing for this reason. Several absorbable carrier materials exist, including bone graft, bioceramic scaffolds (such as calcium phosphate bioceramic and bioactive glass (BAG)), natural (namely, collagen) and synthetic polymeric scaffolds, biodegradable implant coatings (poly(d,l-lactide) (PDLLA)), and hydrogel. Vancomycin and aminoglycosides are the most commonly incorporated antibiotics due to their effectiveness and compatibility with the carriers [4,9,38].

Cement - PMMA

The first use of cement in orthopaedic surgery was in 1960 when Charnley described cement anchoring of a femoral head prosthesis [57]. In 1970, antibiotics were being added to Palacos resins [58]. Antibiotic cement-coated (ACC) nails have been used for over 20 years [59]. Its use in two-stage exchange surgery for PJI has been reported to reduce infection recurrence rates by 7% [60]. It can be moulded into various forms, such as cement strips, beads and implant coating [61]. Furthermore, it can be used as a spacer in large segmental defects and, unlike other carriers, permits weight bearing to improve outcomes [3,18,62]. Antibiotic elution characteristics can be altered as desired, but increasing elution is often balanced against maintaining carrier strength. ‘Home-made’, as opposed to commercially made, hand-mixing, greater surface area and higher antibiotic concentrations increase antibiotic elution [18,38,63].

In terms of prevention, Craig et al. conducted a meta-analysis on the benefit of locally delivered antibiotics at the tissue-implant interface for open tibial fractures treated with intramedullary nailing. Fourteen papers on patients treated with no local antibiotics were compared, with five studies implementing PMMA bead chains impregnated with vancomycin or tobramycin and two studies using gentamicin-coated nails. The risk of infection was significantly reduced with local antibiotic delivery for GA type 3 fractures from 14.4% to 2.4%, with greater benefits found in more severe fractures [64]. A meta-analysis was also conducted by Vargas-Hernandez et al. assessing ACC intramedullary nails in open tibia fractures. They found non-significant reduced rates of non-union and global and deep infections in the treatment group compared to placebo. However, only two studies were included [65]. Morgenstern et al. conducted a meta-analysis on local antibiotic use in open limb fractures. They included six studies implementing PMMA beads but also two testing naked local antibiotics. Significantly lower infection rates with local antibiotic use were reported (4.6% vs. 16.5%, p < 0.001) [66]. Whilst these meta-analyses suggest some clinical benefit, their results should be interpreted with caution as they are limited by the low number of available comparative primary studies with their own risks of bias and heterogeneity. Furthermore, few studies using carriers other than PMMA were included in the meta-analyses. A large matched comparative study would help to confirm findings and gauge the size of an effect. A further use of PMMA cement in FRI prevention is in the first stage of a Masquelet technique for the treatment of critical-size bone defects [67].

For treatment, Qui et al. coated the lateral surface of implants with vancomycin- and gentamicin-impregnated PMMA after a thorough debridement whilst also filling any bony defect. Of the 10 patients included, all achieved union at an average of 5.5 months. One patient with a bone defect had infection recurrence, which was eradicated with further cement application to the defect after the cortical union was reached [67]. ACC nails have also been used for FRI with promising rates of infection control and union in non-comparative studies [59,68-72]. Shyam et al. prospectively studied 23 femoral and two tibial infected non-unions. Infection was controlled in all 20 cases with a bone defect of <6 cm, but none of the five patients with a defect of >6 cm. Only three patients with a bone defect of <4 cm achieved bone union with those who did not undergo nail exchange and bone grafting [68]. Similarly, Bhatia et al. had 95% infection control of tibias with infected non-union and a bone gap of <2 cm, but a union was achieved in 60% with no further procedure [70]. External fixation for infected tibial non-union has comparable rates of secondary procedure (29-62%) and healing rates at final follow-up (92-95%) [71]. ACC nails have been suggested as an alternative to external fixation as they also avoid complications such as pin site infections, pin loosening and adjacent joint stiffness whilst also permitting weight-bearing [70-72]. The most reported complication with this technique is debonding, occurring either at insertion or removal of the nail, which can lead to difficult extraction of the loose parts [73]. Techniques, such as reinforcing cerclage wire, have been recommended to combat this issue [74]. The major disadvantage of PMMA cement with antibiotics is the markedly short duration of elution [75].

Bone Graft

Bone grafts are frequently used in the management of critical-sized bone defects in FRI. Traditionally, bone grafts have been used alone or in combination with antibiotic-impregnated non-absorbable carriers, such as PMMA and cement, as part of a two-stage procedure [76]. More recently, antibiotic-impregnated bone grafts (AIBGs) have been utilised for patients with co-existing infection and osseous defects, such as infected non-union sites [3,77]. A comparative study involving 96 patients with infected tibial non-union demonstrated that AIBGs yielded lower rates of re-infection compared to non-impregnated bone grafts [78]. A further study has reiterated the effectiveness of AIBG in eradicating FRI [79]. AIBGs facilitate bone formation by functioning as a scaffold and introducing biologically active cells and can reportedly store and elute much greater concentrations of antibiotics than other non-absorbable carriers, including PMMA [3]. Moreover, AIBGs do not alter the physical properties of antibiotics, in contrast to bone cement, wherein the polymerisation heat may inactivate the impregnated antibiotic [77]. AIBGs can also be impregnated with a range of antibiotics, which enables targeting of the infecting organisms [3]. For example, studies have shown successful outcomes following the use of autograft bone impregnated with tobramycin, vancomycin and piperacillin [28,78]. However, some evidence suggests that antibiotic impregnation bears an adverse effect on the incorporation of bone grafts, which may result in non-union [79].

Autologous or allogenous bone grafts may be used, depending on the location of the infection, the size of the bone defect and the availability of an internal bone bank [80]. Cancellous bone autografts may be harvested from a variety of sites, including the iliac crest, femur and distal radius [81]. However, autograft harvesting is associated with donor-site morbidity and complications in up to 8.6% of cases, including infection, iatrogenic fracture, wound complications, and sensory deficits [82]. Allograft bone avoids harvesting-related morbidities but does pose an infection risk when used at contaminated sites, both by introducing bacteria and by serving as a sequestrum for bacteria [3]. Moreover, allograft bone lacks the osteoinductive properties of autograft, which may reduce fracture healing and result in delayed or non-union [3]. Consequently, Metsemakers et al. report that allograft bone is not widely used in FRIs associated with bone defects [3].

Current evidence for the use of AIBGs in FRI mostly comprises small case series and retrospective studies. As such, further study is required to investigate the optimal antibiotic agent and dose to eradicate infection and prevent adverse effects on graft incorporation.

Bioceramics

The most frequently used biodegradable ceramic antibiotic carriers are based on either calcium sulphate or calcium phosphate [3]. Evidence suggests superior elution profiles compared to antibiotic-loaded PMMA with a longer duration, leading to decreased infection and biofilm formation [3]. An RCT involving 30 patients with long bone FRIs showed comparable rates of infection eradication and union - but importantly, fewer surgical procedures due to its absorption and lack of removal - following the use of a calcium sulphate and tobramycin preparation compared to antibiotic-loaded PMMA [83]. Another study involving the same bioceramic preparation demonstrated a 91% infection eradication rate in 110 infected fractures following a single-stage procedure [84]. New biodegradable ceramic formulations have since been developed to improve outcomes. For example, a calcium sulphate and gentamicin formulation has been shown to reduce wound healing issues, infection recurrence and re-fracture compared to the tobramycin formulation [3]. A recent prospective study highlighted the benefits of the aforementioned calcium sulphate and gentamicin preparation, including its efficacy in filling dead space and promoting bone remodelling, thereby reducing the re-fracture risk and avoiding the need for bone cement, which would necessitate staged treatment given it is non-absorbable [85].

The effectiveness of BAG in treating chronic osteomyelitis is well evidenced. However, its utility in the treatment of FRI and non-union has gained interest in recent years owing to its antibacterial and osteostimulative properties [18,86]. BAG yields a local bactericidal effect without the addition of a synthetic antibiotic, which avoids the development of bacterial resistance and adverse reactions [86]. In-vitro studies have shown that the antibacterial potency of BAG is similar to that of antibiotic-loaded PMMA [18]. Recent clinical studies have demonstrated encouraging results for BAG in post-traumatic long-bone osteomyelitis. For example, in a study involving 116 patients, of whom 84.5% received non-impregnated BAG as a single-stage procedure, successful eradication of infection was reported in 89.7% of cases [87]. Notably, however, systemic antibiotics were also administered in all patients. Recent in-vitro studies have investigated the use of BAG as an antibiotic carrier to augment its natural antibacterial activity. However, its antibiotic elution efficiency and duration remain unclear due to the heterogeneity of BAG composition and antibiotic choice in studies to date [18]. Further clinical research is required to assess the efficacy of BAG, particularly as an antibiotic carrier, for the treatment of FRI.

Polymers

Collagen is a natural, biodegradable polymer that can be used as an antibiotic carrier [3]. The use of antibiotic-impregnated collagen in the treatment of FRI is scarcely reported, unlike its well-documented application for treating acute and chronic osteomyelitis [80]. A 2011 retrospective review demonstrated the efficacy of gentamicin-impregnated collagen in preventing infection and promoting fracture union in 31 patients with open fractures, in whom 6.5% (n = 2) developed a deep infection and none developed non-union [88]. More recently, an in-vitro study has suggested that a collagen composite comprising osteoinductive and antimicrobial agents would be an ideal pharmaceutical for the regeneration of infected bone defects [89]. Nevertheless, clinical data to assess the efficacy of antibiotic-impregnated collagen in FRI remains lacking.

Several synthetic polymeric scaffolds have undergone in-vitro and in-vivo animal studies to assess their efficacy in treating bone infections with encouraging outcomes [80]. These polymers are thought to be effective antibiotic-delivery systems due to their biodegradability, biocompatibility and minimal toxicity upon degradation. Furthermore, in-vitro studies demonstrate a sustained elution profile, which is an important feature for bone scaffolds [80]. Clinical research in human subjects is needed to substantiate these in-vitro and animal studies’ findings.

PDLLA

Biodegradable implant coating represents a nascent technology in the treatment of FRI. As such, there is currently limited evidence to support its application. To date, a gentamicin-coated tibial nail is the only commercially available PDLLA-coated fracture-related implant. A 2020 systematic review, which included eight studies involving 203 patients, concluded that the gentamicin-coated tibial nail is effective in the prophylaxis of high-risk FRI and treatment of complex non-union cases [90]. Among the 89 patients who underwent revision surgery for non-union (of which 43% were infected), only four patients developed re-infection. In contrast, a recent study found that all five patients who underwent staged complex revision surgery for established FRI failed treatment, suffering ongoing symptoms of deep infection, which required implant removal and further treatment [91]. This study highlighted concerns regarding the use of this gentamicin-coated tibial nail for the treatment of established FRI, especially in view of the high cost. Therefore, antibiotic-PDDLA-coated implants appear to be an effective therapeutic option to prevent FRI in open fractures. However, its efficacy in the treatment of established FRI is controversial and requires further study.

Hydrogel

Hydrogel is a novel and versatile absorbable antibiotic carrier which, due to its consistency, can be applied to implant surfaces as a coating or injected to fill dead space [18]. Compared to PMMA, hydrogel can be mixed with a greater variety of antibiotics and does not inflict thermal damage to the impregnated antibiotic [18]. There is a growing body of evidence supporting its efficacy in the treatment of PJI. However, clinical studies investigating its use in FRI are limited [3,18]. An RCT from 2017 involving 253 patients reported that antibiotic-impregnated hydrogel implant coating successfully reduced the rate of post-surgical site infections after internal fixation for closed fractures without causing any adverse effects [92]. Despite this positive finding, its prophylactic efficacy cannot be extrapolated to the treatment of FRI; the short elution duration and lack of structural integrity (due to its gel-like consistency) may be unsuitable for treating FRI, which typically requires sustained antibiotic elution and the filling of dead space [12]. Therefore, clinical studies focussing on the treatment of FRI are required to clarify these hypothetical considerations.

The advantages and disadvantages of the delivery systems discussed are summarised in Table 1.

**Table 1:** Antibiotic delivery systems summary CLAP, continuous local antibiotic perfusion; FRI, fracture-related infection; PMMA, polymethylmethacrylate; RCT, randomised controlled trial

Antibiotic Delivery	Advantages	Disadvantages	References
Vancomycin powder	Absorbable. Applicable to small spaces. Cheap. RCT with significantly reduced infection rates among high-risk tibial plateau and pilon fractures.	Short therapeutic window. Does not occupy dead space.	[16]
Aqueous gentamicin	Absorbable. Applicable to small spaces. Cheap.	Short therapeutic window. Does not occupy dead space. Limited high-quality research in FRI.	[30]
CLAP system	Continuous therapeutic dosing for chosen period. Implant retention. No carrier.	Does not occupy dead space. Minimally invasive. Evidence from small-scale, non-comparative studies.	[39,54,55]
Cement	Accessibility. Adaptable to a range of geometries and functions. Dead space management.	Not absorbable. Short elution period. Debonding.	[73,75]
Bone graft	Absorbable. Compatible with a variety of antibiotics. Facilitate bone formation. Can store and elute higher antibiotic concentrations (compared to PMMA). Does not alter physical properties of impregnated antibiotic.	Donor site morbidity (autologous grafts).	[3]
Ceramics	Absorbable. Enables bone remodelling. Dead space management. Superior elution profile (compared to PMMA).	Hypercalcaemia. Cost.	[3,85,93]
Polymers	Absorbable. Biocompatible with minimal toxicity upon degradation. Sustained elution profile (in-vitro study).	Evidence mostly based on in-vitro study. Lack of clinical evidence for utility in FRI.	[80]
Poly(d,l-lactide) (PDLLA)	Absorbable.	Limited body of evidence with conflicting results. Expensive. Only one commercially available implant for FRI. Implant is compatible with gentamicin only.	[91,94,95]
Hydrogels	Absorbable. Versatile consistency and application. Does not inflict thermal damage to impregnated antibiotic.	Cost. Lack of clinical evidence. Short elution period. Lack of structural integrity.	[3,12,18]

## Conclusions

There is good evidence for the use of local antibiotics in preventing and treating FRI. At present, various delivery systems - including naked and antibiotic carrier techniques - have primarily been evaluated in small retrospective studies, with the greatest potential benefit seen in preventing infections in high-risk fractures or in treating more severe, resistant infections. Biodegradable antibiotic carriers represent a promising alternative to non-absorbable carriers (i.e., cement), which require surgical removal, with clinical studies involving AIBG and bioceramics demonstrating encouraging outcomes. However, further studies are required to support these findings and to substantiate in-vitro findings for other biodegradable carriers such as BAG, polymers, PDLLA-coating and hydrogels. Despite potential concerns, reports of adverse events in human studies are rare. Additional prospective RCTs are necessary to further clarify the safety and efficacy of various delivery systems for specific fracture patterns.
